# An open source delineation and hierarchical classification of UK retail agglomerations

**DOI:** 10.1038/s41597-022-01556-3

**Published:** 2022-09-03

**Authors:** Jacob L. Macdonald, Les Dolega, Alex Singleton

**Affiliations:** 1 University of Sheffield – Department of Urban Studies and Planning, Sheffield, United Kingdom; 2 Geographic Data Science Lab, University of Liverpool – Department of Geography and Planning, Liverpool, United Kingdom; 3 Consumer Data Research Centre (CDRC), Liverpool, United Kingdom

**Keywords:** Geography, Business, Databases, Economics

## Abstract

Town centres and high streets typically form the social and commercial cores of UK cities and towns, yet, there is no uniform definition of what a town centre or high street is. In this study the spatial delineations of retail agglomerations are generated using open-source data for England, Wales, Scotland and Northern Ireland. The extent and boundaries of these physical retail areas are identified based on the density and connectivity patterns of individual retail units over space. A high resolution hexagonal grid is superimposed over spatial clusters of retail points and a network-based algorithm used to identify mutually exclusive tracts. Agglomerations are then pruned and fine-tuned according to a series of heuristic rules. Our retail agglomerations represent local commerce areas with shopping amenities and are assigned to a hierarchical classification ranking from the largest Regional Centres, Major Town Centres and Town Centres, down to Small Local Centres and Retail Parks. The classification into one of eleven hierarchies is based on a combination of relative rank in the local area and absolute size of retail units within the area. These retail agglomeration boundaries, hierarchical classification and lookups form an open-source spatial data product available for wide use and research implementation.

## Abstract (summary)

**Table:** 

Measurement(s)	Retail Environment
Technology Type(s)	Processed Data
Factor Type(s)	Retail unit locations
Sample Characteristic - Environment	usage of an environment for retail activity
Sample Characteristic - Location	United Kingdom

## Background & Summary

Town centres and high streets typically form the social and commercial cores of UK cities and towns and are characterized by clusters of retail alongside other supporting businesses and leisure offerings. Yet, there is no uniform definition of what a town centre or high street is and there are a number of terms such as retail centre or retail agglomeration that are often used interchangeably. Moreover, there are also many other destinations where agglomerations of retail occur that fall outside of central locations; such as those spanning arterial roads, local neighbourhood centres or out of town and planned retail parks^1^. It has long been recognised that retail agglomerations are part of a complex system that constantly evolves, and as such, the spatial extent of such agglomerations can expand or contract over time^2^. Depicting the spatial extent of different retail agglomerations at a national scale in a rigorous and systematic way is pivotal for a better understanding of the relationship between use of retail space and changing consumer behaviour^3^. In particular, capturing systematic metrics of retail centres, monitoring their economic performance and providing new insights through retail analytics requires an up to date and robust set of retail centre boundaries^3,4^.

The computation of such definitions is a long standing issue. UK town centre boundaries were developed by Thurstain-Goodwin and Unwin (2000) and adopted by the Department of Communities and Local Government (DCLG) in 2004^5^. Their approach involved generating kernel density estimation (KDE) surfaces employing various socio-economic variables such as building density, diversity of building use and tourist attraction^6^; thus capturing a wider definition of town centre function beyond purely retail. Since then, new forms of retail centre occupancy data from both commercial (e.g. Local Data Company) and open source (e.g. Valuation Office Agency or Open Street Map) destinations have become available alongside new methods and techniques enabling their analysis at scale. Such developments have enabled the creation of national retail agglomeration boundaries that are more up to date and often with better granularity. A former endeavour to capture spatial extent and hierarchy of UK retail centres employed Local Data Company (LDC) occupancy data and used a bespoke density-based spatial cluster (DBSCAN) algorithm^3,7^, however the data used was not in open access and the method developed not easily replicable due to being calibrated to that specific dataset^8^.

This work presents the UK retail agglomerations data product, developed using a replicable and tractable geographic data algorithm. Through the combination of open-source, accessible, data and a transparent methodology, we delineate the most up to date and robust spatial extents of retail agglomerations for the whole of the UK, representing delineated retail and leisure service locations. Data are integrated from the Valuation Office Agency (VOA) and Open Street Map (OSM), with the resulting definitions formed through heuristic rules where well defined, localized, high retail density is observed.

A total of 6,423 retail agglomerations are identified across the country and are assigned a position within a hierarchical classification, grouping them according to their size and function in the region, county, local authority and urban area. The agglomerations are sorted into one of eleven groups ranging from larger Regional Centres or Major Town Centres to the smaller Market Towns, District Centres, Local Centres or Retail Parks. The classifications were ascribed using both relative size ranking across administrative geographies and absolute size of units within the centre. These distinctions among the network are an important first classification since larger density retail spaces may attract consumers from a longer, more regional, distance, while smaller localized centres which are more widespread throughout cater primarily to the local resident population^6^.

Identifying and mapping the variation in these spaces has a range of important policy implications from local planning, employment distributions, or economic value and vitality. As the future of high streets and consumption areas in the UK increasingly plays a role across many sectors of the economy and local workforces, the distribution of these physical spaces is important to consider in tandem with other socio-economic conditions or measures of neighbourhood built characteristics^9,10^. Our spatially detailed and comprehensive delineation of retail spaces across the UK provides an important benchmark for future works seeking to better understand, among other areas, retail geography and economic performance of these important space across various spatial scales.

This research data product provides retail centre boundaries for all four countries across the UK: England, Wales, Scotland and Northern Ireland using a consistent definition and delineation of retail areas based on common national and accessible data products. The final open source data product is available for wide research and applied use, further providing a replicable methodology and framework for continued work and application to other contexts.

## Methods

The delineation of retail centres is based on the density of individual retail point locations over space. Where there are spatial clusters of retail, contiguous tracts of small area geometries are used to build corresponding self-contained unique centres for all four countries in the UK – England, Wales, Scotland and Northern Ireland. A hierarchical classification of centres is generated alongside the delineated geographies to broadly classify centres according to size and function across eleven nested groups ranging from *Major Town Centres* to *Small Local Centres*.

### Retail location data and cleaning

The first stage in delineating the spatial extent of retail agglomerations was to assemble a UK database of geocoded retail store locations. The most extensive and openly accessible data varied by country. For England and Wales, the VOA non-domestic ratings registry has been used by previous studies to for identifying retail locations^11–13^. These data served as the primary source of retail location data; whereas in Scotland and Northern Ireland where VOA data are not available, these were replaced with OSM retail-specific point data^14,15^.

The most recent comprehensive VOA assessment of non-domestic properties was completed in April, 2017; which aims to identify each hereditament (property) and an ascribed rateable value which is used in taxation. Other supplementary attributes include an address, a typology of the property use, along with structural characteristics such as size and proportions of the property used for different purposes (e.g. warehouse versus the shop front). The full registry includes around two million non-domestic property assessments, however vacant and inactive units are not explicitly identified, and locations extracted from the registry are taken to represent the population of actively trading retail units.

A subset of retail and leisure properties were identified from the top 800 most common categories and include a range of retail amenities including shops, convenience stores, salons, cafes, stalls, pubs, take away and food premises, and leisure amenities including gyms, cinemas, museums, and nightclubs. The property types used to identify “retail” units are included in Table 1. From this set, we remove any potentially identified units which include offices, petrol stations or related non-retail premises and are left with 617,953 retail specific units across England and Wales.

**Table 1:** VOA Hereditament Category Types Selected.

VOA Retail Categories			
Amusement Arcade	Fitness Centre/Studio	Library	Sports Centre
Art Gallery	Food Court	Market Stall	Store
Barbers Shop	Funeral Parlour	Museum	Superstore
Betting Shop	Gallery	Nightclub	Swimming Pool
Bingo Hall	Gymnasium/Gym	Pet Grooming Parlour	Takeaway
Bowling Alley	Hairdressing/Beauty Salon	Pharmacy	Tattoo Studio/Parlour
Bureau De Change	Health & Fitness Club	Post Office	Theatre
Coffee Shop/Tea Room	Hotel	Pub/Bar	Treatment Room
Casino	Kiosk	Restaurant	Veterinary Surgery
Cinema	Foodstore/Supermarket	Retail Unit	Yoga Studio
Dance Studio/School	Launderette	Retail Warehouse	
Dental Surgery	Leisure Centre	Shop	

Note: Including variants of the category names in the data, for e.g. “Gallery”, “Gallery and Premises”, “Art Gallery”, “Gallery and Shops”, etc.

**Fig. A1:**
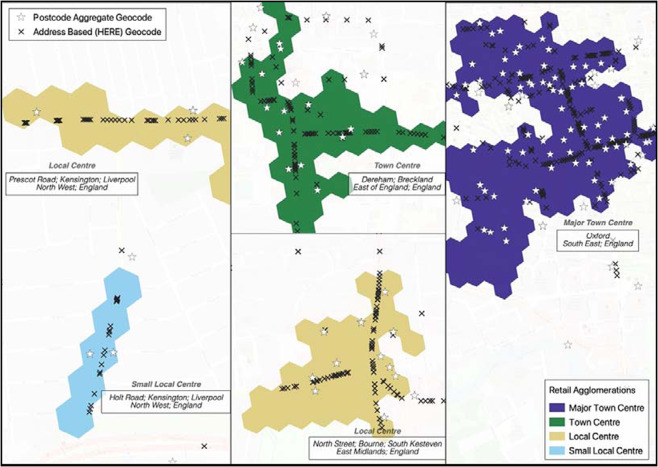
Postcode and Address Level Geocoding.

Each VOA hereditament is supplied with an address, which is then geocoded using the HERE geocoder API. These address locations were cleaned and validated by comparing the obtained address level coordinates with the coordinates of the postcode alone extracted from the Office for National Statistics Postcode Directory. In the UK, there are around 13 addresses on average within each postcode, so a postcode derived geocode is less accurate, only approximating an address. Figure A1 of the Appendix highlights the benefits of a more precise geocoding in delineating the boundary extents. Geocoding errors were identified where there was a large difference between the postcode and address locations (over 5 kilometres), most commonly attributed to discrepancies in the address format or name. In such cases the retail units were dropped from the analysis so that the clusters accurately reflected the true density of local points and not postcode approximate aggregates or incorrect matches. Of the full retail sample, 23,793 units were dropped with a total of 594,160 units remaining with address level geocoding.

For Scotland and Northern Ireland, where VOA ratings are not available, and for further supplementing the VOA data in England and Wales, retail-based points were extracted from the ***R*** OSM API^16^. Retail units were defined from a set of *key* and *value* combinations that are coded onto the OSM spatial features. The list for what constitutes the set of retail-based points are in Table 2 and include any element with a “shop” key identified, and twelve subset (*values*) of “amenity” points including pubs, restaurants and leisure amenities. A point for each retail (shop or amenity) unit was returned, which form the core basis of retail properties from which agglomerations are delineated in Scotland and Northern Ireland. In total, 15,715 shops and 12,580 (of the subset) amenities are identified via the OSM data in Scotland (28,295 total retail units), while 3,122 shops and 2,470 amenities are identified in Northern Ireland (5,592 total retail units).

**Table 2:** OSM Retail Category Types Selected.

OSM Retail Categories (*keys and values*)			
***key*** = ***“shop”***			
*value* = <ALL>			
***key*** = ***“amenity”***			
*value* =			
Bank	Cafe	Marketplace	Post Office
Bar	Fast Food	Nightclub	Pub
Bureau de change	Food Court	Pharmacy	Restaurant

The additional data for England and Wales further allowed us to compare the retail point locations extracted from this set of data versus those obtained from the VOA non-domestic property registry. By comparison, 186,836 shops and 143,499 amenities are identified in England and Wales via the OSM data for a total of 330,335 OSM retail units compared to the 594,160 VOA retail units used for these regions. We use the VOA points as our base from which retail agglomerations are delineated using small area building blocks. OSM retail points data is used as a supplement in England and Wales where there is no VOA coverage in a corresponding local (small) area.

### Retail centre delineation

To define the spatial extent of retail agglomerations we adopted H3 geometries (https://eng.uber.com/h3/). This is a hexagon-based grid system that has global coverage, and was designed by Uber as a flexible and hierarchical set of units that could be utilised within multiple locations for the aggregation of mass point data – in their case, the aggregation of GPS tracks from the use of their tracked vehicles. Retail centres are built by identifying the tracts of granular (H3) hexagon spatial units which have retail point clusters, and through their spatial contiguity and connectedness.

The advantage of H3 is its availability at multiple spatial scales (sizes of hexagon) and global coverage, enabling replication of these methods for other contexts where similar data are available. The ability to assign retail unit points to a hierarchical index of hexagons increasing in size, and with consistent coverage, greatly reduces potential biases from the chosen spatial unit. The granularity with which retail centres are built is particularly advantageous when compared with alternatives such as administrative or statistical boundaries, for example, which often vary greatly in size and shape across an entire country. These units further enable the efficient calculation and comparison of local density measures using retail counts. The base hexagonal units chosen have an average area around 2000 m^2^ with an approximate radius of 50 metres (H3 unit size 11). The ascription of these unique H3 IDs to each agglomeration also facilitates wider compatibility with ancillary data that also utilise these geometry.

We start by identifying a comprehensive population sample of H3 units where any VOA or OSM retail points are found, or in the case of Scotland and Northern Ireland using only OSM points. When conducting any retail counts or aggregates it is important to separate the VOA and OSM units to avoid overcounting. Given the different data sources, it is not possible to identify duplicate locations across the sets of points.

We remove any H3 spatial units where only a single retail unit (of either VOA or OSM variety in England and Wales) was recorded within them. Given the size of spatial units, roughly corresponding to a segment of street or an intersection, a singular VOA or OSM retail point indicates a very low retail density area such as a free standing store, leisure or service unit. Pre-pruning these small areas removes a significant clutter of low density hexagons. In the case that these single unit hexagons are in the interior of a retail agglomeration, they are filled in during the post-cleaning stage – with all relevant retail counts and statistics recalibrated for the full extent. When at the boundary, allocating the entire hexagon due to a singular point would not be necessary to meaningfully extend the existing agglomeration.

The resulting overlay of hexagon geometries creates an exhaustive coverage of retail point clusters across the UK. From this set we apply a graph algorithm and make use of the efficiency of H3 indices to identify mutually exclusive and contiguous tracts of hexagons^17^. Neighbouring hexagons are easily identified and joined into unique groupings using the H3 indexing, avoiding the need for complex spatial operations. Each cluster is assigned an individual ID and represents the first iteration of retail agglomerations.

When the returned agglomerations were inspected, there were instances where the representations might be deemed a poor match to the real world structure of retail within the locations that they purported to represent. This was most noticeable in agglomerations where the address geography mapped onto constrained locations whereas the full extent of the retail area might be much larger. For example, an out of town retail park or shopping centre might have a large extent covering multiple builds and parking areas, however may only have a single address location for all occupiers of the site. In such instances, although the count of the retail locations may be high, these may be aggregated into a very limited number of H3 polygons where the geocoded locations resided. To correct such issues, we drew strength from further OSM data by extracting land use polygons that were labelled with categories specifically classified as retail (*key = “land use”; value = “retail”*). H3 geometries were then derived to cover the extent of the polygons.

In the case of England and Wales, the additional padding of retail land use is only appended when adjacent to existing H3 units where there are VOA retail units. We thus exclude any large self-contained retail land use contiguous tracts of OSM padding where there was no identified retail units. Comparatively in Scotland and Northern Ireland, where the analysis comprised entirely OSM derived retail point locations, these large self-contained land use tracts are not excluded, even when no existing retail points (or respective hexagons) are located directly adjacent.

**Fig. 1:**
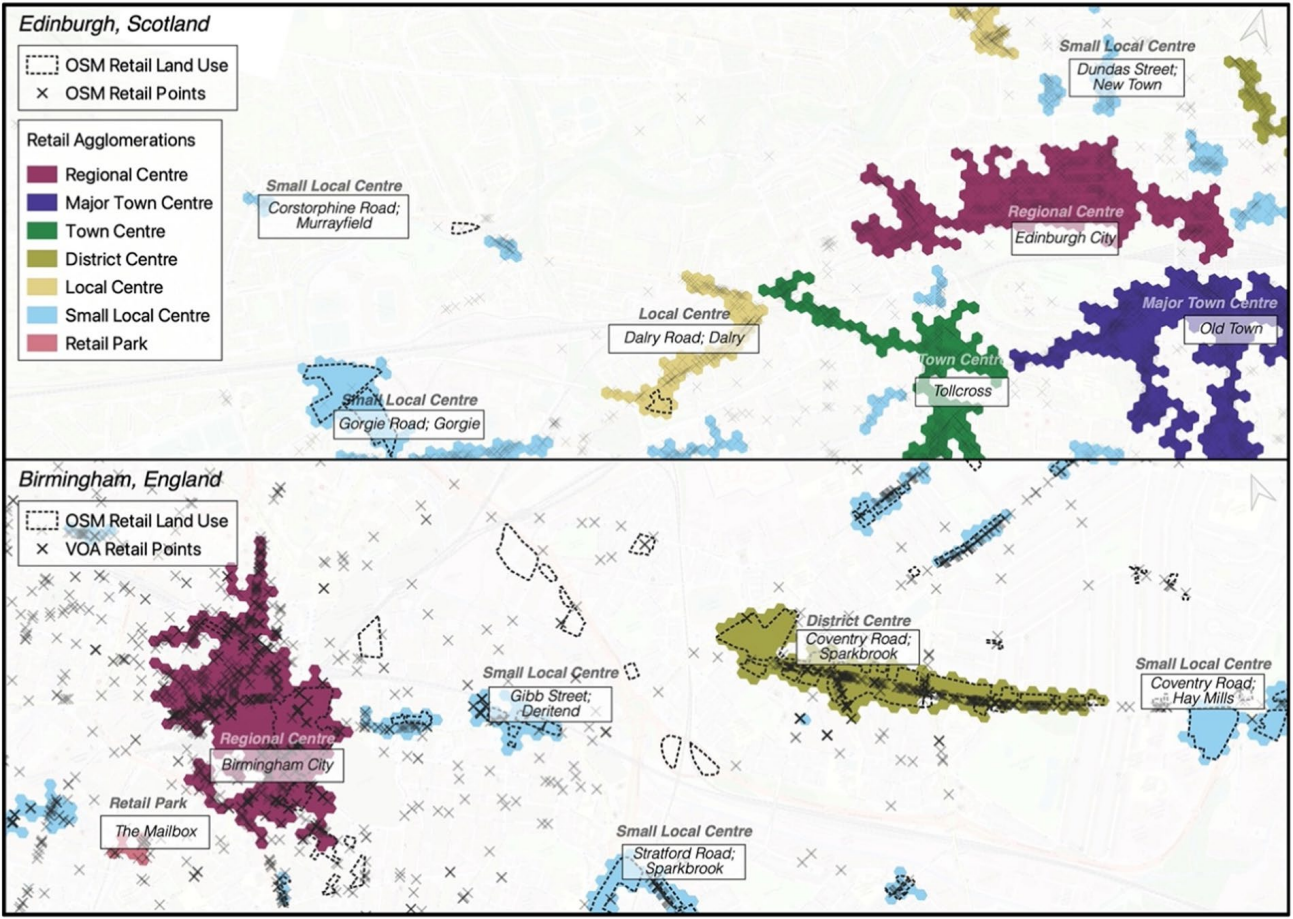
Retail Centre VOA & OSM Foundations with OSM Padding.

Figure 1 highlights the benefit of this padding in either case by extending the natural retail boundary beyond the location of single points representing retail areas. Respective OSM retail points in Edinburgh and VOA retail points in Birmingham are mapped alongside retail agglomerations and OSM retail land use padding. We see, in the case of England and Wales, that purely OSM retail land use tracts are not included unless adjacent to specified retail unit tracts. The emphasis here is on using accurately geocoded address locations to provide the most appropriate delineation, whereas isolated OSM tracts without any identified retail may either suffer from low coverage, or be capturing different typologies of retail (e.g. car showrooms, industrial estates). Additional padding can be seen in the District Centre and Small Local Centre along Coventry Road in Birmingham – extending the delineated points-based hexagons to include more appropriate extents of the shopping area.

An advantage of H3 geometry is that this includes an indexing system that makes it efficient to locate and extract the reference index for contiguous or nested spatial units. We prune the exhaustive set of retail centres to include only those with at least two hexagon units and at least ten overall retail unit points contained within them, a threshold used by Pavlis *et al*. (2018) and based on UK retail planning policy^18^. In Scotland and Northern Ireland, this criteria is relaxed specifically to allow for the larger self-contained OSM tracts to be included (those without underlying retail unit points). In England and Wales, agglomerations with less than fifteen VOA retail points and ten OSM retail points are removed from the set, while for Scotland and Northern Ireland the condition is based on ten OSM points. These threshold values for England and Wales are in line with comparable high street delineation methods from the Ordnance Survey (OS)^19^.

From the subset of removed hexagon units based on the above rules, we re-introduce those which are composed of a single isolated hexagon unit if the cell had a minimum of ten VOA (from which we have address level geocoding) retail units. This exception is made to capture isolated island retail centres with highly dense activity; such as small, compact local neighbourhood shopping parades. Just under 150 unique isolated hexagons with over ten retail units are included back into the set, coming from England and Wales.

Isolated (singular) hexagons with nine or fewer retail units are excluded. For the vast majority of retail space of interest, these small potentially significant areas are captured by association with OSM retail land use padding and adjacency to other retail spaces. This padding further captures cases when a small number of singular point retail locations do not adequately represent the relatively larger physical retail space (e.g. Supermarkets). Smaller and more isolated retail areas are thus outside the scope of our defined retail agglomerations.

While the above algorithmic approach does not require any conditioning based on local context, certain features may be overlooked and require post-processing and validating by human experts. For example, we make no consideration of rivers, canals or natural boundary breaks (e.g. train tracks) which may in reality create a de-facto split between areas which are otherwise computationally linked. Conversely, the algorithm may split a centre into two based on a gap in retail units that is just larger than the approximate diameter of a hexagon – the more common issue.

A series of consultations were used to refine and make bespoke alterations to the identified retail agglomerations. An online interactive web map was developed on the CARTO platform (https://carto.com) and shared for wider consultation. Local domain experts, academic research groups and the wider geospatial mapping community in the UK academic network were approached for a series of ground truthing exercises. Based on their local knowledge the stakeholders were asked to provide feedback on any retail agglomerations that they are familiar with in their local area, in particular whether any boundaries needed to be merged, split, removed or added.

Through this ground truthing process, 6 agglomerations were removed, 3 were split and 36 were merged together. The final set of retail agglomerations after amendment comprised a total of 6,423 areas: 5,611 in England, 341 centres in Wales, 392 located in Scotland and 79 in Northern Ireland.

### Retail centre naming and hierarchical classification

Although our classification is derived with open data and methods, a hierarchical (count based) classification and technical validation were conducted on the retail centre delineations supplemented with secured data from the Local Data Company (LDC), available via controlled access through the ESRC Consumer Data Research Centre (CDRC)^20^. This allowed us to use more recent data, account for vacant units and cross validate against the VOA and OSM retail totals. LDC produce a national rolling retail survey capturing detailed information on location, category, vacancy and tenure; alongside further information on which retail units are considered to be within “retail parks”. Large (out of town) shopping centres and designer outlets are identified from a bespoke listing from online sources. Following other hybrid open and commercial data driven projects^21^, data were accessed through the CDRC (https://data.cdrc.ac.uk) and code is deposited to enable reproducibility within their secure setting where these commercial data can be accessed.

Retail agglomerations across the UK were organised into a hierarchy of categories ranging from the largest of Regional Centres to the Small Local Centres. The retail counts within each area are calculated for both the LDC and VOA data. While LDC data is limited in Scotland and Northern Ireland, the detail and scope of retail units surveyed in available areas is detailed, particularly with regards to vacant units and more timely coverage, provide a range of additional insights. From both the LDC and VOA retail counts we take the maximum of the two to use during the size-based classification. In Scotland and Northern Ireland OSM data is used in place with the same procedures of taking the maximum where there are multiple sources.

**Fig. 2:**
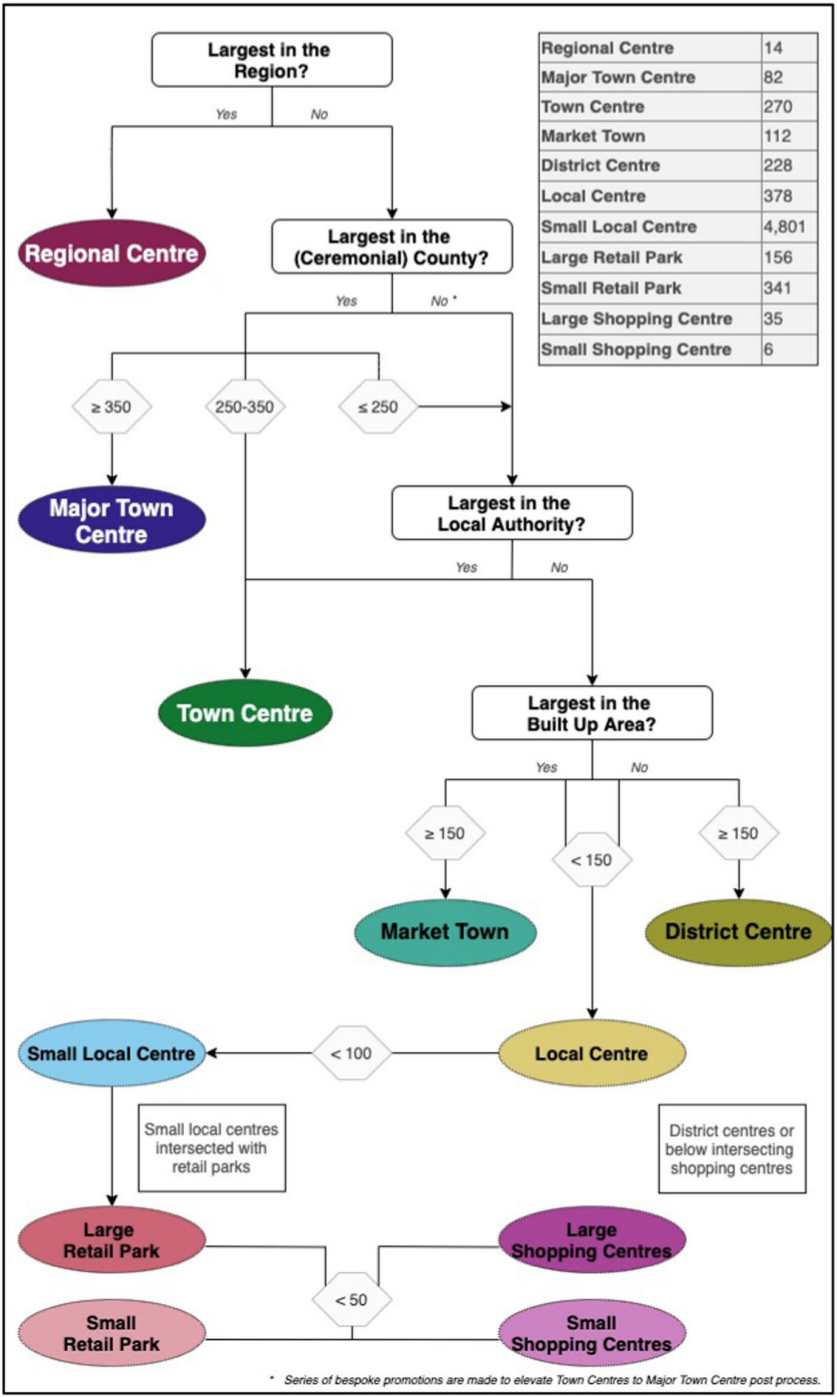
Retail Agglomeration Hierarchical Classification.

These broad categories to which retail agglomerations are assigned are meant to capture the relative local size of each area across the country and are further used to facilitate naming conventions. Figure 2 outlines the classification process through which areas are assigned a place in the hierarchy. Broadly, retail agglomerations are classified according to their absolute retail unit count along with the relative ranking of their count within a series of geographic administrative units. The three largest agglomeration groups, for example Regional Centres, Major Town Centres, and Town Centres, are all classified as such for being the highest ranking count (size) within the national geographic administrative boundaries of the Region, the Ceremonial County, or the Local Authority District respectively. We further impose absolute retail count criteria of 250 units upon these to ensure a minimum size is met for higher ranking hierarchies. Table A1 of the Appendix summarizes the different hierarchies.

Towards the bottom of the hierarchy are the localized Market Towns and District Centres. While both have a minimum of 150 retail units, Market Towns are the highest ranked in the Built up Area and potentially serving as more of a hub. Local and Small Local Centres form the core of accessible retail agglomerations to local residents across the country. Retail outlets and (out of town) shopping centres specific are identified using the external sources and are further classified according to their overall size with 50 retail units serving as a cut-off for Small and Large Retail Outlets and Shopping Centres respectively. In total, eleven categories of retail centres are classified across five general hierarchy levels. Table 3 shows this distribution across countries after a series of bespoke promotions or demotions among the larger Regional, Major and Town Centres. The hierarchy of London is further updated according to the London Plan retail network classification.

**Table 3:** Distribution of Retail Agglomerations by Hierarchy and Region.

	Regional Centre	Major Town Centre	Town Centre	Market Town Centre	District Centre	Local Centre	Small Local Centre	Retail Park*	Out of Town Shopping*
**England**	10	73	238	98	222	326	4,188	422	36
***London***	*1*	*10*	*33*	*0*	*84*	*79*	*858*	*53*	*4*
**Wales**	1	3	17	10	4	33	244	28	1
**Scotland**	2	6	17	4	2	19	292	47	3
**N**. **Ireland**	1	0	0	0	0	0	77	0	1
**United Kingdom**	14	82	270	112	228	378	4,801	497	41

*Includes both Small and Large areas.

Out of all retail agglomerations, London is home to approximately 17.5% of them, 12% of the Regional and Major Town and Town centres. The density of retail agglomerations in London is predominantly among the Local and Small Local Centres. The count of these centres in London (79 and 858 respectively) are comparable and somewhat higher to the count of these types in the entire north of England (North East and North West) which has 61 Local Centres and 846 Small Local Centres.

Along with the unique identifier associated to each agglomeration through the graph algorithm process and the general hierarchical classification based on relative size, each retail agglomeration is assigned a more conventional unique name identifying the locale. The set of names are drawn from local streets, geographic administrative areas and the OS Open Names database^22^. The naming convention is meant to convey street, neighbourhood and city detail across these three levels. Given the size of Regional, Major Town and Town Centres, we use only neighbourhood and city distinction as many of these areas are made up of numerous dominant streets.

We extract information on the conventional names from numerous sources. At the street level the most common street address among all retail units in the agglomeration is extracted from the set of retail VOA points, or using the LDC equivalent if missing. The primary street (or both primary and secondary if two streets both have over 31% of the distribution) is used as the lowest level of naming amongst Market Town Centres, District Centres, Local Centres and Small Local Centre.

The second level, representing the name of the broader neighbourhood, is extracted from a series of OS Open Name categories. In the first instance, retail centres overlapping directly with the exact point location at where these names are spatially represented are attributed the name. Where no direct intersection takes place, the nearest conventional name via Euclidean distance is used.

Lastly, Local Authority District names are used at the third level to represent the broader areas where the retail agglomeration sits. In the rare case where a cluster sits on the border of two Local Authorities, the tie is broken by the percentage in spatial overlap. Each name is further appended with the Region and Country convention to facilitate focusing on these sub-national geographies.

## Data Records

The retail agglomerations data product and accompanying information are provided online through the Consumer Data Research Centre (CDRC) open data repository available through^23^: https://data.cdrc.ac.uk/dataset/retail-centre-boundaries.

The retail centre boundaries and related information are provided across a series of files which can be matched together using the unique ID. Variable descriptions and metadata for each of the files are included respectively below.

1. ***Retail_Boundaries_UK.gpkg***
  The core boundary file is in a GeoPackage format (requiring spatial software to read). This file includes the spatial boundaries of all retail agglomerations located across the UK – England, Wales, Scotland and Northern Ireland.
  *Variables:*

**Table:** 

*RC_ID:*	Retail centre reference ID
*RC_Name:*	Retail centre conventional name
*Classification:*	Hierarchical classification based on retail counts
*Country:*	Country of origin: England, Wales, Scotland or Northern Ireland
*Region_NM:*	Internal England region name (+ Scotland, Wales and Northern Ireland)
*H3_count:*	Count of H3 hex geometries making up the tract
*Area_km2:*	Total size (km^2^) of retail centre
*Retail_N:*	Count of retail points within the tract
  For each retail centre the accompanying reference ID and conventional names are included, along with the hierarchical classification and broad location and size. A count of retail units are included only for the largest retail agglomerations which have over 100 units, corresponding to 1,153 centres. Two measures of physical size are included, the area in squared kilometres and the count of H3 hexagon geometries which make up the agglomeration. Given the consistent size of hexagon units, this second measure can also be used to explore measures of retail densities.
2. ***Classification_hierarchy.csv***
  A lookup table providing the full name and brief description of the hierarchical classification. A hex colour code with respective visually accessible colour palette is provided to replicate maps and figures from this report. Table A1 of the Appendix replicates this table.
  *Variables:*

**Table:** 

*Classification:*	Hierarchical classification based on retail counts
*Summary:*	Brief summary of classification category
*Hex_Col:*	Hex colour code to replicate colour scheme from Fig. 2
3. ***OA_Lookup_table.csv***
  A spatial lookup table between the generated retail centres and small area geographies is provided to facilitate merging in external spatial data at different geographies. Output Area (OA) and Workplace Zone (WZ) lookups are provided for England and Wales, along with the respective OA delineations for Scotland and Northern Ireland.
  The lookup table provides a list of all small area geographies which intersect the respective retail agglomeration and their proportionate overlap. Each retail centre may thus overlap in different proportions across multiple small areas. Respective indicators are included, where available, to identify whether the population weighted centroid is within the retail centre.
  *Variables:*

**Table:** 

*RC_ID:*	Retail agglomeration reference ID
*OA_CD:*	Small area geographic code
*OA_Area:*	Physical size (m2) of area overlap between the OA and retail centre
*Coverage:*	Percent of OA overlap with respective retail agglomeration
  An equivalent file for WZ areas is provided for England and Wales. Data files are open access and available for download from the online repository. Each individual record refers to a specific geographic area representing the delineated boundary of high-clustered retail areas along with the accompanying metadata and information.

## Technical Validation

In addition to the expert stakeholder consultation we implemented to garner feedback on the delineation of our retail agglomerations, a further technical validation was also developed to make comparisons with an external definition of high streets that is supplied by the OS, the national mapping agency in Great Britain^24^. For this external validation, we compare the coverage of our retail agglomeration boundaries relative to the location and size of respective OS High Streets. We focus on assessing the physical geographic match between the two different data sets, along with the count of retail unit locations which fall within the respective boundary definitions.

The OS High Streets data are comprised as a line geometry for the street alongside the corresponding retail locations and a bounding box for the spatial extent of the high street identified. For this validation we use the bounding box around each line to represent the areas which are most comparable to our retail agglomerations. Further, high streets from the OS are for Great Britain only and thus there is no comparison with Northern Ireland.

**Fig. 3:**
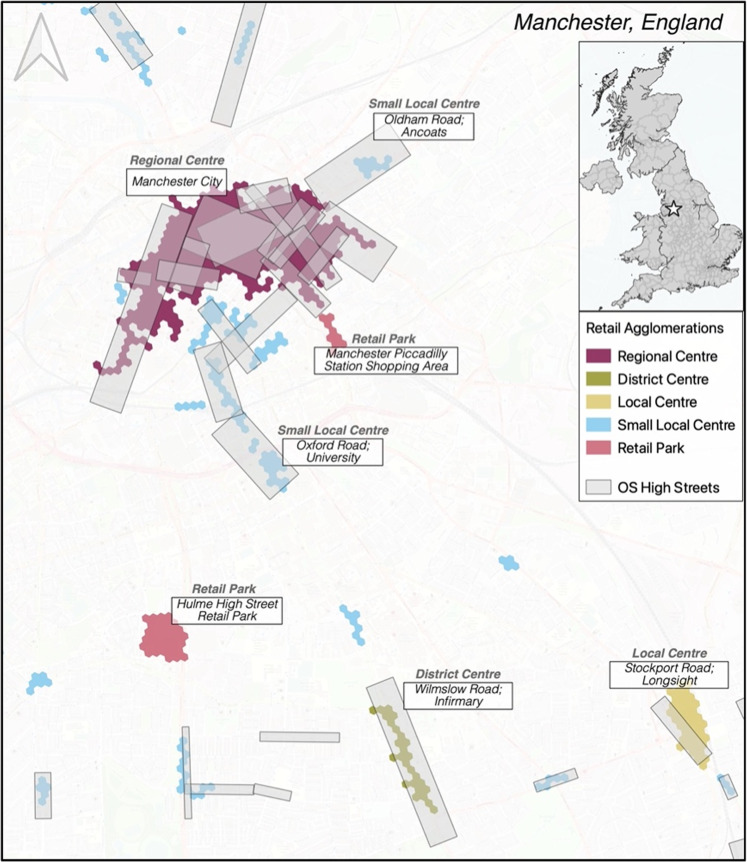
Retail Units Count and Comparison.

It is important to consider that the underlying methodology of our developed retail agglomerations and that of the OS high streets are fundamentally different. While the retail agglomerations delineate a set of mutually exclusive areas representing clusters of retail, the OS high streets are designed to identify individual commercial-focused streets themselves and those buildings that these comprise. As such, multiple overlapping streets from the OS definitions may overlap our generated retail agglomeration. Figure 3 contextualizes the difference between OS High Streets and our identified retail agglomerations in the case of central Manchester, in the North West of England. Vis a vis the OS product, our developed retail agglomerations might be argued as providing a less granular delineation of the retail space, however the objective here was to establish wider and mutually exclusive self-contained centres of retail activities. As such, these centres of activity are the combination of potentially multiple streets and segments with concentrations of retail.

We first compare the overall total area of physical land occupied by the respective high streets and retail centres. Using the auxiliary 2020 LDC data we further count the number of retail units within the agglomerations and the OS high streets. The goal is to provide a comparison of our retail agglomerations where they best intersect with local high streets. LDC data provides a purpose built third-party retail count targeted in these areas and useful for comparing the two retail boundary sources. Table 4 shows the comparison in coverage and size distribution – including the land area covered, the count of respective (OS) high streets or our retail agglomerations, and a comparison of respective areas with at least 100 (LDC) retail units. We break down the analysis by country to similarly capture the underlying differences between how the centres were developed in each area.

**Table 4:** Retail Agglomeration Count and Size Comparison.

	OS High Streets			Retail Agglomerations		
	*Size (km*^2^*)*	*% Overlap*	*Count*	*Size (km*^2^*)*	*% Overlap*	*Count*
**England**	295.1	48.3	6,136	308.4	46.1	5,611*
*100+*	*127.9*	*50.1*	*1331*	*165.8*	*38.6*	*1432*
**Wales**	13.5	44.4	376	14.9	40.2	341
*100+*	*3.91*	*46.5*	*56*	*6.62*	*27.5*	*101*
**Scotland**	22.1	38.8	457	26.4	32.6	392
*100+*	*10.2*	*44.4*	*110*	*13.2*	*34.5*	*89*
**N**. **Ireland**	—	—	—	6.3	—	79
*100+*	—	—	—	*0.51*	—	*1*

Notes: % Overlap refers to the percent of all High Street physical space that are covered by the corresponding Retail Agglomeration space (and vice versus).

*Excluding areas where there is no coverage from LDC retail points.

As expected, there are more OS high streets when compared to the delineated retail agglomerations which, as seen in Fig. 3, can represent a combination of individual streets. In terms of their physical space however, both the high streets and retail centres are much closer in coverage. Although the extents and delineation of the areas differ substantially, we still see between 30 – 50% overlap of these physical spaces on one another across the UK, highest in England with lower correspondence of space in Scotland. When looking at the larger retail spaces, there is a higher correspondence of high streets being ‘captured’ (covered) by the retail agglomerations. Further, if we extend a buffer of 100 meters around the retail agglomerations we capture a significantly larger portion of delineated OS High Street areas, accounting in part for the differences in geometries used. The correspondence of the enhanced buffer zone increases to 79.7%, 79.5%, and 66.6% respectively for England, Wales and Scotland (compared to 48.3%, 44.4% and 38.8%).

**Fig. 4:**
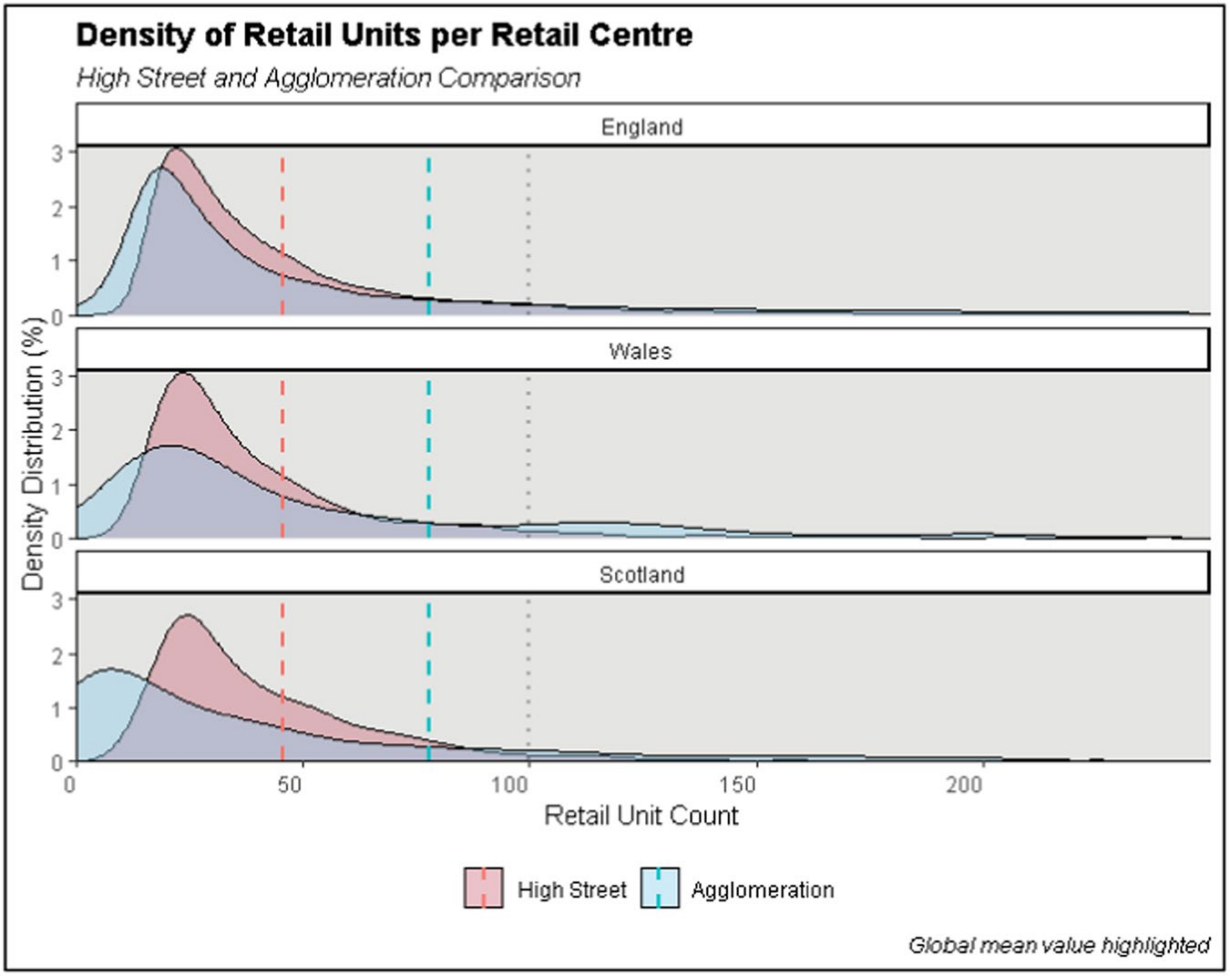
Retail Centre and High Street Density Distributions.

A primary difference between the two data products is the bulk of small centres (under 100 units) which are in the OS high streets dataset yet combined under a larger retail agglomeration through our definition. Comparable to Table 4, the correspondence of space for these small areas ranges from 16 – 30% compared to the larger agglomeration. With significantly more small singular high street areas (see e.g. Figure 3), with smaller counts of retail and leisure specific units, the distribution of OS areas is skewed more towards the lower tail compared to larger retail areas (Fig. 4). As seen with the physical coverage of space, the agglomerations at the higher end of the hierarchy are more closely comparable than the smaller areas. Comparing this distribution of sizes to the comparison of the overall totals in Table 5, however, show that while the distributions may be quite different, the total coverage of retail units captured is comparable.

**Table 5:** Retail Units Count and Comparison.

	Baseline	LDC Retail			OS Retail*	OS – LDC Corr.
	*Total*	*Total*	*Retail Aggl*.	*OS High Streets*	*Total*	
**England**	559,245 *(VOA)*	567,195	404,336	423,910	282,081	0.782
			*(71.3%)*	*(74.7%)*		
**Wales**	34,915 *(VOA)*	26,013	17,857	18,183	15,422	0.793
			*(68.7%)*	*(69.9%)*		
**Scotland**	28,295 *(OSM)*	57,697	28,875	35,607	20,278	0.762
			*(50.0%)*	*(61.7%)*		
**N. Ireland**	5,592 *(OSM)*	5,288	1,621	—	—	—
			*(30.7%)*			

*Refers to OS-based retail and leisure units used in the OS High Streets and the relevant correlation with patterns of LDC retail units in the same space.

We continue using LDC data for the comparison of the number of retail units in Table 5 to look at overall differences in retail size (count of units). Table 5 further highlights the original total count of retail units within the retail agglomerations as coming from the original sources – VOA data for England and Wales, and OSM data for Scotland and Northern Ireland. While the underlying source of retail location data differs – OS retail units for the high streets and the combination of VOA, OSM and LDC data for our retail agglomerations, we can see that in terms of general coverage and retail units captured, the outputs are quite comparable. The relative difference in retail units as conducted through the commercial LDC survey captured in between the two definitions is small compared to the number and distribution of total across the countries, and further a large percentage (up to 70% in England and Wales) of retail units fall within either of the delineated retail spaces.

The definition of retail and leisure units which the OS high street definition use as their base is also provided. Looking at the postcode level, the set of unique postcodes attached to retail units from the VOA set contained within the retail agglomerations in England and Wales is 61,475 compared to the 66,567 unique postcodes from the set of OS-based units – which includes Scotland as well. Overall, we see a general correspondence of common retail and space between the two definitions. While the core definition of what is considered retail space differs substantially between the LDC retail units survey and the OS definition of retail and leisure buildings, there is still however strong correlation, just below 0.8, between the OS high street units and the respective count of LDC survey units within those high streets.

## Figures and Tables

**Fig. A2:**
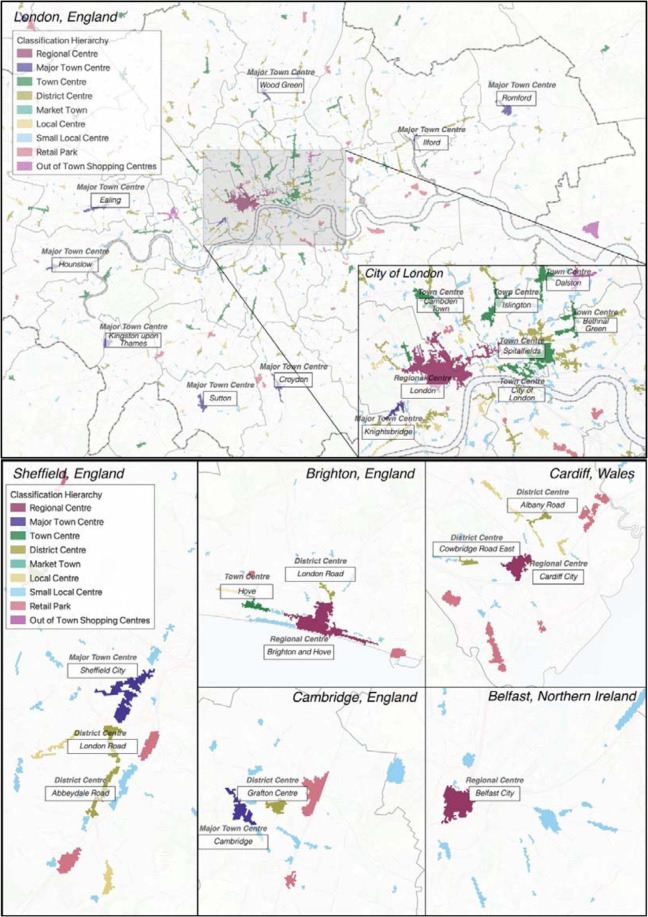
Example Retail Agglomerations Across the UK.

## Data Availability

The CDRC repository hosts both the output data files along with the R code and scripts used to clean the input data and reproduce the analysis – where based on openly available data. The series of files and code are available online through:https://data.cdrc.ac.uk/dataset/retail-centre-boundaries, https://github.com/ESRC-CDRC/Retail-Centre-Boundaries. Scripts are written and the analysis conducted in *R* version 4.1.1^25^. Core packages for replicating the generation of the dataset, including the spatial and network operations, include: *h3*^26^, *sf*^27^, *data.table*^28^, *tidyverse*^29^, *tidygraph*^30^, *igraph*^17^, and *osmdata*^16^.

## Appendix

**Table A1:** Description of Hierarchical Classification.

Classification	Summary	Hex. Colour ID
Regional Centre	Largest retail centre in the broader administrative region	#882255
Major Town Centre	Significantly large retail centres. Largest in the ceremonial county with a minimum of 350 retail units	#332288
Town Centre	Large retail centre (largest in the local authority or within the ceremonial county) with at least 250-350 units	#117733
Market Town Centre	Largest centre in the respective built up area with at least 150 retail units	#44AA99
District Centre	Not the largest centre within a built up area, but has at least 150 retail units	#999933
Local Centre	Retail centres with under 150 units	#DDCC77
Small Local Centre	Retail centres with under 100 units	#88CCEE
Large Retail Park	*Small Local Retail Centres* which are designated as Retail Parks with 50-100 units	#CC6677
Small Retail Park	*Small Local Retail Centres* which are designated as Retail Parks with under 50 units	#E0A1AB
Large Out of Town Shopping Centre	Larger out of town shopping centres and outlets	#AA4499
Small Out of Town Shopping Centre	Smaller out of town shopping centres and outlets with under 50 units	#CD83C1
